# Ship Fever

**Published:** 1847-07

**Authors:** 


*Ship Fever.*—The last No. of the New-York Annalist gives the following account of the prevalence of the ship fever among the emigrants of the city of New-York:—

" Emigrant ships crowded with sick and starving passengers continue to arrive daily in our harbor. Our hospitals are crowded to overflowing, and tents, barns, &c., are needed to contain the sick, until sheds can be constructed to receive them. The end of this crying evil, it is impossible to foresee. A foolish and infuriated mob, a few days ago, burned the buildings formerly used as nurseries on the Long Island Farms, which were intended to receive the convalescent from Staten Island. The deaths by Typhus, during the week ending from May 22 to May 29, were 49."

The Academy of Medical Science, have adopted the following resolution.

"*Resolved*, That a Committee of eleven Fellows be appointed by the Chair, to visit the public and private Hospitals in which it abounds, for the purpose of investigating the subject of said Fever, with special reference to its causes, symptoms, treatment, and pathology, and report to this Academy as soon as the objects for which it is appointed shall have been accomplished."

The Annalist states that the committee have already commenced their labors.

The disease, as we learn from the newspapers, prevails, also, extensively among the immigrants at Philadelphia, Boston, and Quebec. A few cases are said to have occurred at this city.

